# miR-511 Deficiency Protects Mice from Experimental Colitis by Reducing TLR3 and TLR4 Responses via WD Repeat and FYVE-Domain-Containing Protein 1

**DOI:** 10.3390/cells11010058

**Published:** 2021-12-25

**Authors:** Shafaque Rahman, Jolien Vandewalle, Patricia H. P. van Hamersveld, Caroline Verseijden, Olaf Welting, Aldo Jongejan, Pierina Casanova, Sybren L. Meijer, Claude Libert, Theodorus B. M. Hakvoort, Wouter J. de Jonge, Sigrid E. M. Heinsbroek

**Affiliations:** 1 Tytgat Institute for Liver and Intestinal Research, Amsterdam Gastroenterology Endocrinology and Metabolism, Amsterdam University Medical Centers, Location AMC, University of Amsterdam, 1105 BK Amsterdam, The Netherlands; s.rahman@amsterdamumc.nl (S.R.); h.p.vanhamersveld@amsterdamumc.nl (P.H.P.v.H.); c.verseijden@amsterdamumc.nl (C.V.); o.welting@amsterdamumc.nl (O.W.); pierina.casanova@gmail.com (P.C.); t.hakvoort@amsterdamumc.nl (T.B.M.H.); sigrid.heinsbroek@gmail.com (S.E.M.H.); 2 Department of Biomedical Molecular Biology, Ghent University, 9052 Ghent, Belgium; JolienM.Vandewalle@UGent.be (J.V.); claude.libert@IRC.VIB-UGent.be (C.L.); 3 VIB Centre for Inflammation Research, VIB, 9052 Ghent, Belgium; 4 Bioinformatics Laboratory, Department of Epidemiology and Data Science, Amsterdam University Medical Centers, Location AMC, University of Amsterdam, 1105 AZ Amsterdam, The Netherlands; a.jongejan@amsterdamumc.nl; 5 Department of Pathology, Amsterdam University Medical Centers, Location AMC, University of Amsterdam, 1105 AZ Amsterdam, The Netherlands; s.l.meijer@amsterdamumc.nl; 6 Department of Surgery, University of Bonn, 53113 Bonn, Germany

**Keywords:** miR-511, intestinal inflammation, mouse, wdfy1, TLRs

## Abstract

Antimicrobial responses play an important role in maintaining intestinal heath. Recently we reported that miR-511 may regulate TLR4 responses leading to enhanced intestinal inflammation. However, the exact mechanism remained unclear. In this study we investigated the effect of miR-511 deficiency on anti-microbial responses and DSS-induced intestinal inflammation. miR-511-deficient mice were protected from DSS-induced colitis as shown by significantly lower disease activity index, weight loss and histology scores in the miR-511-deficient group. Furthermore, reduced inflammatory cytokine responses were observed in colons of miR-511 deficient mice. In vitro studies with bone marrow-derived M2 macrophages showed reduced TLR3 and TLR4 responses in miR-511-deficient macrophages compared to WT macrophages. Subsequent RNA sequencing revealed Wdfy1 as the potential miR-511 target. WDFY1 deficiency is related to impaired TLR3/TLR4 immune responses and the expression was downregulated in miR-511-deficient macrophages and colons. Together, this study shows that miR-511 is involved in the regulation of intestinal inflammation through downstream regulation of TLR3 and TLR4 responses via Wdfy1.

## 1. Introduction

MicroRNA’s (miRNAs) are short (18–24 nucleotides in length), endogenous, noncoding single-stranded RNA’s that regulate gene expression by controlling stability and translation of protein-coding mRNA [1]. Post-transcriptional regulation by miRNAs affects many processes, including cell development, cell differentiation, tissue homeostasis, and activation of the immune system [2,3,4]. miR-511 is a miRNA embedded within intron 5 of the CD206 gene (MRC1), referred to as the macrophage mannose receptor [5,6]. After transcription and cleavage, the pre-miRNA is formed, which is transported to the cytoplasm where through further cleavage a miR-511-3p-miR-511-5p duplex is formed. Subsequently, miR-511-5p is known to be degraded, and miR-511-3p is the bioactive mature strand derived from both human and mouse pre-miR-511 and the human miR-511-3p sequence is conserved in both species [6]. This strand is highly conserved amongst mammalian species, and its levels correlate with CD206 expression, which is particularly high in anti-inflammatory, wound healing, and tumor-associated macrophages [6,7,8]. 

The role of miR-511 in diseases has been reported where several studies have found anti-tumor effects of miR-511 in human cancers, including breast cancer [9], lung adenocarcinoma [10], colorectal cancer [11], and osteosarcoma [12]. miR-511 was also shown to contribute to neuronal differentiation [13] and limit allergic inflammation in a mouse model of asthma [14]. Furthermore, miR-511 was shown to inhibit endotoxemia and experimental hepatitis through the regulation of TNFR1 in mice [15]. Hundreds of other miR-511-3p potential targets are found in databases (Targetscan, Diana microT), such as Rho-associated coiled-coil containing protein kinase 2 (ROCK2), zinc finger proteins x-linked, 518B, activin A receptor type IIB (Acvr2b) and others in mice and zinc finger proteins; ROCK2 and transcription factor 4 and activating enhancer binding protein 2 beta are some targets found in humans [6], where most of the genes in mice and humans are involved in biological processes and targets such as ROCK2 and zinc finger protein 518B, and Acvr2b is conserved in both mice and humans [6]. We previously showed that CD206 deficient mice also lack miR-511, which affected macrophage toll-like receptor (TLR)-4 ligation responses. These mice showed reduced anti-microbial responses and dampened intestinal inflammation compared to wild-type (WT) mice [16]. Other studies also reported a role for human miR-511 in TLR4 signaling, but the exact mechanism remained unclear [5,17]. 

To investigate the exact role of miR-511 in intestinal inflammation, we studied the effect of miR-511 deficiency in a mouse Dextran Sodium Sulphate (DSS) colitis model. DSS disrupts the epithelial barrier, which makes it a good model to study innate immunity and gut barrier function in the colon [18,19]. In this study, we show that miR-511-deficient mice develop less severe colitis compared to its control. RNA sequence analysis on bone marrow-derived macrophages (BMDMs) identified WD repeat and FYVE-domain-containing 1 protein (Wdfy1) as one of the few differentially expressed proteins between WT and miR-511-deficient macrophages. Our study also shows downregulation of Wdfy1 protein in colons and bone marrow-derived macrophages in miR-511-deficient mice. Wdfy1 is reported to be a critical adapter molecule of the TLR 3/4 signaling pathway [20], and WDFY1 deficiency is related to impaired TLR3/TLR4 immune responses [21,22]. TLR3 signaling is associated with antiviral immune responses as well with the pathogenesis of inflammatory disease [23,24], and TLR4 is shown to be upregulated during inflammation and two single nucleotide polymorphisms in the TLR4 gene are associated with Ulcerative colitis and Crohn’s disease [25,26]. We further found that miR-511 bone marrow-derived macrophages stimulated with TLR3 and 4 agonists indeed displayed a reduced pro-inflammatory response compared to WT macrophages. In conclusion, this study shows that miR-511 plays an essential role in intestinal inflammation by regulating TLR3 and TLR4 responses via Wdfy1 in mice. 

## 2. Materials and Methods

### 2.1. Mice 

C57Bl/6N (WT) mice and C57BL/6 miR-511-deficient (A kind gift from Prof. Claude Libert) mice were housed and maintained under specific pathogen free conditions in individually ventilated cages on a 12/12 h light/dark cycle in 20 °C  ±  2 °C and 55% humidity with *ad libitum* food and water access in our animal facility, Animal Research Institute (ARIA) at the Academic Medical Center in Amsterdam. Additional adult C57Bl/6N mice were bought from Charles River Laboratory (Maastricht, The Netherlands) for in vitro experiments. All animals were acclimatized in ARIA before experiments. Mice were kept and handled according to guidelines of the Animal Research Ethics Committee of the University of Amsterdam. The Animal Research Ethics Committee approved the mouse experiments (DMO276AA).

### 2.2. DSS-Induced Colitis

Mice aged between 10 to 14 weeks were used for DSS colitis experiments. Male and female mice were housed in separate cages and they were distributed in a random manner between groups. Two DSS colitis experiments were conducted to assess clinical scores and for the isolation of intestinal cells. 2% (*w*/*v*) DSS (TdB Consultancy, Uppsala, Sweden) was added to the drinking water (Innovive, Thermo Fisher Scientific, Leiden, The Netherlands) and refreshed every day for 7 days. Bodyweights were recorded daily and mice were euthanized with CO_2_ at the end of the experiment. Colon lengths and wet colon weights were recorded. Colon weight per cm was used as a disease parameter. Disease activity index was determined based on body weight loss score, where a score of 0 indicates less than 1% weight loss, 1 = 1–5% weight loss, 2 = 5–10%, 3 = 10–15% and the highest score of 4 is given to a weight loss of more than 15%, stool consistency score (0 = normal consistency, 1 = loose droppings, 2 = loose stool, colon filled with feces, 3 = loose stool, feces near caecum, 4 = empty bowel) and stool bleeding scores ranging from 0 which indicates no bleeding to a score of 4 for grossly/bloody stool [27]. Colons were longitudinally divided in two parts, where one part was used for histology and the other part was snap frozen to isolate RNA. 

### 2.3. Histology

For routine histology, formalin-fixed hematoxylin- and eosin-stained tissue sections were microscopically evaluated by an experienced pathologist in a randomized blinded fashion. A score from 0 to 4 was given to colons as an indication of severity of inflammation based on the characteristics, namely: affected area (0 = 0%, 1 = 1–10%, 2 = 10–25%, 3 = 25–50%, 4 = >50%), number of follicles (0 = 0–1 follicles, 1 = 2–3 follicles, 2 = 4–5 follicles, 3 = 6–7 follicles, 4 = >7 follicles), level of edema (0 = absent, 1 = minimal, 2 = mild, 3 = moderate, 4 = severe), level of fibrosis (0 = absent, 1 = minimal, 2 = mild, 3 = moderate, 4 = severe), level of erosion/ulceration (0 = 0%, 1 = 1–10%, 2 = 10–25%, 3 = 25–50%, 4 = >50%), level of crypt loss (0 = 0%, 1 = 1–10%, 2 = 10–25%, 3 = 25–50%, 4 = >50%), and granulocyte and monocyte infiltration (0 = Normal, 1 = minimal increase, 2 = mild increase, 3 = moderate increase and 4 = severe increase). Colitis histology index was calculated as the total score of the above mentioned categories and as described previously [28,29,30,31]. Representative stained colon tissue section pictures were taken using an Olympus BX51 microscope (Olympus Nederland BV, Leiderdorp, The Netherlands).

### 2.4. Fluorescence Activated Cell Sorting of Colonic Lamina Propria Cells 

To isolate single cells for fluorescence activated cell sorting, colons were opened longitudinally, washed in (*v*/*v*) phosphate-buffered saline (PBS) (Fresenius Kabi, Huis Ter Heide, The Netherlands)) and cut into 0.5 cm pieces. Colon pieces were further processed as previously described [16]. Briefly, after five washes in Hanks’ balanced salt solution (HBSS) (Thermo Fisher Scientific, Bleiswijk, The Netherlands) with 2% (*v*/*v*) Fetal Bovine Serum (FBS) (Bodinco BV, Alkmaar, The Netherlands)), tissue pieces were first incubated for 20 min at 37 °C in calcium/magnesium-free HBSS supplemented with 2% (*v*/*v*) FBS and 5 mM EDTA (Fisher Emergo B.V, Landsmeer, The Netherlands) with constant shaking. Thereafter, tissues were washed in cold (*v*/*v*) PBS and cut into 0.1 cm pieces with further incubation at 37 °C for 40 min in calcium/magnesium-free HBSS supplemented with 2% (*v*/*v*) FBS, 5 mg mL^−1^ Liberase Blendzyme (Roche Diagnostic Nederland BV, Almere, The Netherlands), and 10 μg mL^−1^ DNAse (Roche Diagnostic Nederland BV) under vigorous shaking. Once digestion was completed, cell suspension was passed through a 100 μm cell strainer (Corning BV, Amsterdam, The Netherlands). Cell suspensions were first incubated with Fc block (BD Pharmingen, Breda, The Netherlands) and incubated for 10 min on ice. Subsequently, cell suspensions were stained with PerCP-conjugated anti-CD11b (Biolegend, Uithoorn, The Netherlands), APC-conjugated anti-Ly6C (eBioscience, Vienna, Austria), APC-Cy7-conjugated anti-CD45 (Biolegend), PE-Cy7-conjugated anti-MHCII (Biolegend), Fluorescein isothiocyanate-conjugated anti-Ly6G (Biolegend), PE-conjugated anti-CD64 (Biolegend), and Alexa700-conjugated anti-CD11c (eBioscience). Cells were washed three times with (*v*/*v*) PBS with 2% (*v*/*v*) FBS. Cells were sorted using FACS cell sorter, LSR Fortessa (BD Biosciences, Erembodegem, Belgium), and different immune cell populations (monocytes, macrophages, neutrophils and dendritic cells) were collected in (*v*/*v*) PBS with 2% (*v*/*v*) FBS.

### 2.5. BMDM Culture and Stimulations 

Bone marrow cells were isolated from tibiae and femurs of adult male and female C57BL/6N and miR-511-deficient mice and were cultured for 7 days at 37 °C with 5% CO_2_ in RPMI-1640 containing: L-glutamine, 25 mM HEPES (Thermo Fisher Scientific), 10% (*v*/*v*) FBS (Bodinco BV), 1% (*v*/*v*) penicillin-streptomycin (Fisher Emergo B.V) and 15% (*v*/*v*) L929-cell conditioned culture medium (ATCC, Manassas, VA, USA) as source of macrophage colony-stimulating factor (M-CSF). Subsequently, cells were detached with 4 mg/mL lidocaine-HCL (Sigma-Aldrich Chemie BV, Zwijndrecht, The Netherlands) supplemented with 5 mM EDTA (Merck, Darmstadt, Germany). Cells were plated at 1 million cells/mL on a 6-well tissue culture plate (VWR International BV, Amsterdam, The Netherlands). Cells were skewed into M2 macrophage states with 40 ng/mL of Interleukin 4 (IL-4) (Tebu-Bio, Heerhugowaard, The Netherlands) for two days [16]. M2 differentiated bone marrow-derived macrophages were then stimulated with 100 ng/mL lipopolysaccharide (LPS; Bio-Connect, Huissen, The Netherlands), 10 Zymosan particles/cell (Sigma-Aldrich Chemie BV) or 10 ng/mL Poly I:C (Invivogen Europe, Toulose, France), respectively, for 24 h after which supernatants and cells in Tripure isolation reagent (Sigma-Aldrich Chemie BV) or RLY lysis buffer (Bioline ISOLATE II mini kit, GC Biotech, Alphen aan den Rijn, The Netherlands) were collected for further analysis. Also, following similar protocol M0 macrophages from breeding colonies of C57Bl/6N (WT) mice and C57BL/6 miR-511-deficient mice were used for miR-511 detection.

### 2.6. mRNA, cDNA and qPCR Analysis 

Colonic tissues were homogenized in Tripure isolation reagent with stainless steel beads (Qiagen Benelux B.V, Venlo, The Netherlands) for 15 min using a tissue homogenizer (Qiagen Benelux B.V). mRNA was then isolated according to the manufacturer’s protocols. Bioline ISOLATE II mini kit was used for RNA clean up in accordance to manufacturer’s protocol. 

RNA from BMDM was isolated using the Tripure reagent (Sigma-Aldrich Chemie BV). RNA from BMDMs for sequencing was isolated with the Bioline ISOLATE II mini kit according to manufacturer’s protocol and RNA quality was checked by Tapestation 2200 (Agilent technologies Netherlands B.V., Amstelveen, The Netherlands). cDNA from both colonic tissues and BMDMs was synthesized with 0.19 µg/µL of Random Hexamer primers (Promega, Leiden, The Netherlands), 10 µM Oligo dT primers (Thermo Fisher Scientific), 1 mM deoxyribonucleotide triphosphate (dNTPs) (Thermo Fisher Scientific), 1x RT-buffer (Thermo Fisher Scientific), 5 U/µL Revertaid Transcriptase (Thermo Fisher Scientific) and 1 U/µL Ribolock RNAse Inhibitor (Thermo Fisher Scientific). Quantitative polymerase chain reaction (qPCR) was performed on a LightCycler 480 II (Roche Diagnostic Nederland BV) or Bio-Rad CFX96 (Bio-Rad Labratories BV, Lunteren, The Netherlands) using SensiFAST SYBR No-ROX (GC Biotech BV, Waddinxveen, The Netherlands). Liver tissues were homogenized in Tripure reagent similar to the colonic tissues) using a tissue homogenizer. To detect miR-511 expression, microRNA from BMDMs and liver lysates was isolated using miRNeasy mini kit from Qiagen according to protocol (Qiagen Benelux B.V). For miR-511 detection, a protocol for running custom RT and preamplification pools on custom TaqMan array microRNA cards was used (Life Technologies Europe, Bleiswijk, The Netherlands). mTNF-α, mIL-6, mIL-10, mCXCL10, mMCP-1, mTLR4, mCD206 and mWdfy1 mRNA levels were measured and analyzed using LinRegPCR software [32]. Glyceraldehyde-3-phosphate dehydrogenase (GAPDH) and Cyclophilin were used as housekeeping genes for colon samples, whereas Eukaryotic Translation Elongation Factor 2 (EEF2) and ribosomal protein-large-P0 (RPLP0) were used for BMDMs. All the housekeeping genes and TNF-α, IL-6, IL-10, CXCL10, CD206 and Wdfy1 primers were synthesized by Sigma-Aldrich Chemie BV and primers for MCP1, TLR4, miR-511-3p and housekeeping gene Taqman miRNA Assay mmu-miR-140 were synthesized by Life Technologies Europe. The primer sequences are listed in Table 1. 

### 2.7. Cytokine Measurements

In supernatant of BMDM stimulations, protein concentrations of TNF-α, IL-6, and IL-10 were measured with a mouse inflammation kit, BD Cytometric Bead Assay (CBA; BD Bioscience, Vianen, The Netherlands) according to manufacturer’s instructions, with the exception that samples were diluted 3 to 4 times. Samples were acquired on the flow cytometer, LSR Fortessa. 

### 2.8. RNA Sequencing of BMDMs 

RNA quality was assured using the Agilent 2200 Tapestation, using only samples with a RIN score of ≥8. mRNA was isolated and converted into cDNA with the KAPA mRNA HyperPrep Kit (Roche Diagnostic Nederland BV, Almere, The Netherlands), whereupon the cDNA was prepared for sequencing on the HiSeq4000 at the Core Facility Genomics, Amsterdam UMC in a 50 bp single-ended fashion to a depth of 40 M per sample. The raw reads were checked for quality using FastQC (v0.11.15), trimmed for adapter sequences using Trimmomatic v0.32 and aligned to the *mus musculus* genome (GRCm38v93) using HISAT2 (v2.1.0). Counts were obtained using HTSeq using annotation fromEnsembl v94 [33]. Duplication rates were inspected using dupRadar v1.0.0. Genes with more than 2 count-per-million reads in 2 or more of the samples were kept. For normalization, the weighted trimmed mean of M-values (to the reference) is used (TMM (edgeR)) [34]. Genes were reannotated using BiomaRt and Ensembl (v100). The count data was transformed to log2-counts per million (logCPM) using voom, estimating the mean-variance relationship. Differential expression was assessed using a Bayes moderated t-test using the linear model framework from the limma package [35] using the Benjamini–Hochberg false discovery rate to correct for multiple testing of the resulting p-values. Homologene v68 was used to map the Entrez Gene IDS from mus musculus to human to be able to perform a gene set enrichment analysis against the human genesets from MSigDB v7.0 (H,C1,C2,C3,C5,C6,C7) [36,37]. For this gene set enrichment analysis we used the CAMERA [38] function from the limma package, with inter.gene.corr = 0.01. Analysis was performed using Rv4.0.0 and Bioconductor v3.11 [39,40]. 

### 2.9. Westernblot

Bone marrow-derived M2 macrophages were lysed in RIPA buffer (0.15 M NaCl, 0.05 M TRIS pH 7.5, 1% NP-40, 0.5% DOC 0.1% sodium dodecyl sulphate (SDS) pH 8.0), followed by the protein quantitation based on bicinchoninic acid (BCA) protein assay (Thermo Fisher Scientific). The protein samples were run on 10% SDS-polyacrylamide gel electrophoresis (PAGE) gels and transferred to a nitrocellulose membrane (Brunschwig Chemie BV, Amsterdam, The Netherlands). The membranes were further blocked in 5% (*w*/*v*) bovine serum albumin (BSA) (Sigma-Aldrich Chemie BV) solution in 1X (*v*/*v*) Tris buffered saline (TBS) (blocking buffer) for two hours at room temperature. Thereafter, incubating the membrane with Wdfy1 antibody (Biorbyt, Huissen, The Netherlands) (1:1000) in the blocking buffer overnight at 4 °C. The membranes were then washed 3 times with 1X (*v*/*v*) TBS-Tween-20 (T) (VWR International BV, Amsterdam, The Netherlands), incubated with HRP conjugated secondary antibody (1:2000, Dako Netherlands BV, Amstelveen, The Netherlands) in blocking buffer for 1 h. Membrane was also incubated with the loading control, GAPDH antibody (Sigma-Aldrich Chemie BV) at 1:1000 dilution for 2 h, washed 3 times with 1XTBS-T, and incubated with HRP conjugated secondary antibody (1:2000, Dako Netherlands BV) for 1 h in the blocking buffer. Protein expression was detected with Lumilight (Roche Diagnostic Nederland BV).

### 2.10. Immunohistochemistry 

Immunohistochemistry was done to visualize WDFY1 protein in colon sections of WT and miR-511-deficient control and DSS induced mice and in human. Non-IBD control (colorectal carcinoma), IBD (Crohn’s) inflamed and non-inflamed colon sections from male adult participants were used. The specimen collection was approved by the biobank review committee under biobank number 178#A201470 and written informed consent was obtained from all study participants. The paraffin embedded sections were deparaffinized in xylene and gradually rehydrated in (*v*/*v*) ethanol gradient to PBS followed by antigen retrieval in sodium citrate buffer, pH 6.0 for 20 min at 98 °C and 30 min blocking in 10% normal goat serum (R&D Systems, Abingdon, UK). The slides were incubated overnight at 4 °C with primary antibody (rabbit polyclonal anti-WDFY1, 1:400) in blocking buffer. The slides were blocked in 3% (*v*/*v*) hydrogen peroxidase (H_2_O_2_) in PBS for 20 min followed by a 30 min incubation with secondary antibody (anti-rabbit polyclonal HRP, Brightvision BV, Almere, The Netherlands). Staining was visualized after a 4 min incubation with chromagen substrate diaminobenzidine liquid plus (DAB, Dako Netherlands BV). The sections were finally counter stained with hematoxylin and mounted. Images were processed using the Olympus BX51 microscope. 

### 2.11. Statistical Analysis 

Graphpad Prism 9.1.0 (GraphPad Software, La Jolla, CA, USA) was used for statistical analysis. All data are expressed in mean ± SEM. Individual t-test were used to assess statistical significance of difference where *p* is ≤0.05 (*), 0.01 (**), 0.001 (***) or 0.0001 (****).

## 3. Results

### 3.1. miR-511 Deficiency Affects Macrophage Responses to Microbial Stimuli

**Figure 1:**
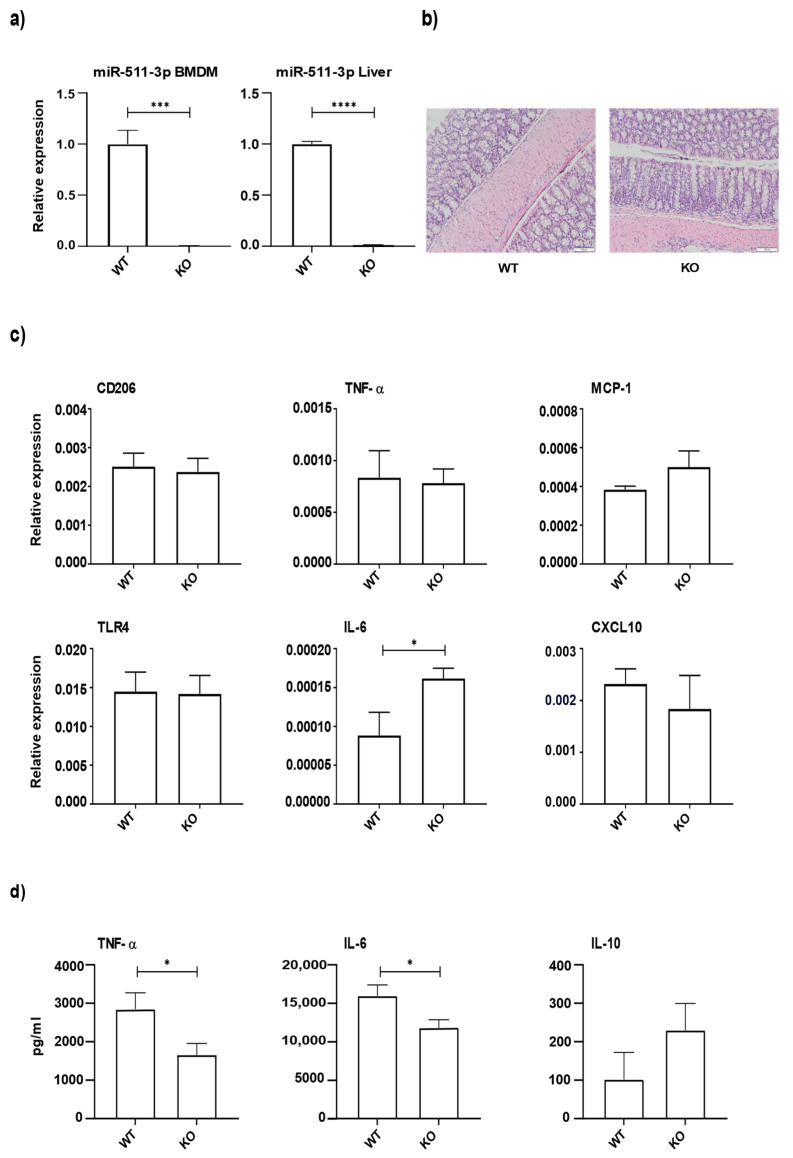
The effect of miR-511 deficiency on macrophage LPS responses. (**a**) Validation of the miR-511-3p bioactive strand in BMDM and liver tissues by qPCR. N = 4 for both wild-type (referred to as WT in figures) and miR-511-deficient (referred to as KO in figures) mice. (**b**) Representative hematoxylin and eosin stained histology picture of colons from WT and miR-511-deficient mice, 10× magnification. (**c**) mRNA relative expression of CD206, TNF-α, MCP-1, TLR4, IL-6, and CXCL10 in colon homogenates of WT- and miR-511-deficient mice by qPCR. N = WT = 4, KO = 5. mRNA levels were normalized for their respective reference genes. (**d**) Cytokine response (TNF-α, IL-6 and IL-10) in BMDM after LPS stimulation by CBA. N = WT = 5–6, KO = 6. Individual values are expressed as mean and standard error of the mean. Statistical differences were tested by independent *t*-test, where a *p*-value of <0.05 was considered to be significant. * *p*-value < 0.05; *** *p*-value < 0.001; **** *p*-value < 0.0001.

Previously we suggested that miR-511-3p affects macrophage TLR4 responses and ameliorates intestinal inflammation [16]. We further investigated the exact effect of miR-511 deficiency on intestinal inflammation using miR-511-deficient mice. As expected miR-511-deficient mice lacked miR-511-3p in BMDMs and liver tissue (Figure 1a). Colons of miR-511-deficient mice showed no infiltration of granulocytes/monocytes, ulceration, fibrosis etc. as shown by histology (Figure 1b). No differences were observed in relative mRNA expression of CD206, TNF-α, MCP-1, TLR4, and CXCL10 in colons of WT and miR-511-deficient mice (Figure 1c). Interestingly, a slight but significant upregulation of IL-6 expression was seen in miR-511-deficient mice compared to WT (Figure 1c). Since we previously found that knockdown of miR-511-3p reduces macrophage lipopolysaccharide (LPS) responses we tested if macrophages from miR-511-deficient mice were also affected in their LPS responses. miR-511-3p is highly expressed in M2 derived macrophages [6] therefore, BMDM from miR-511-deficient and WT mice were cultured and M2 differentiated macrophages were stimulated with LPS (Figure 1d). These macrophages showed a significant reduction in TNF-α and IL-6 response and no significant effect was seen on IL-10 response upon LPS stimulation compared to WT M2 macrophages (Figure 1d). In contrast, no significant effect was seen on TNF-α, IL-6 and IL10 mRNA levels of these macrophages (supplementary Figure S1a). Together, our data show that miR-511-deficient mice have healthy colons but macrophage function is affected. 

### 3.2. miR-511-Deficient Mice Are Protected against DSS-Induced Colitis 

**Figure 2:**
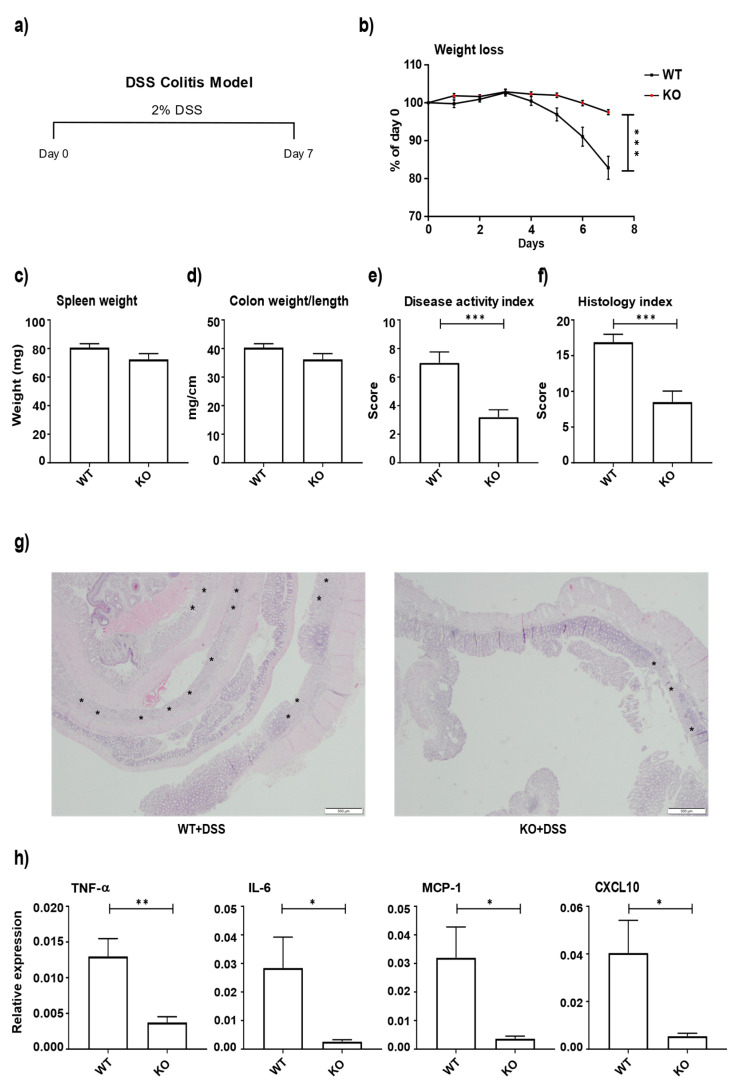
miR-511 deficiency ameliorate DSS-induced colitis. (**a**) A schematic presentation of DSS colitis in mice. (**b**) Bodyweight changes between WT and miR-511-deficient mice after colitis induction. (**c**) Spleen weights. (**d**) Colon weight/length ratio. (**e**) Disease activity index score. (**f**) Colitis histology index score. (**g**) Representative histology picture of colons from WT and miR-511-deficient mice, 2× magnification, asterisk represents erosion and ulceration (**h**) mRNA expression of TNF-α, IL-6, MCP-1, and CXCL10 in colon homogenates of all groups by qPCR. N = 9 WT and 10 miR-511-deficient mice for (**b**–**f**,**h**). mRNA levels were normalized for their reference genes. Individual values are expressed as mean and standard error of the mean. Statistical differences were tested by independent *t*-test, where a *p*-value of <0.05 was considered to be significant. * *p*-value < 0.05; ** *p*-value < 0.01 and *** *p*-value < 0.001.

To address the effect of miR-511 in intestinal inflammation, both WT and miR-511-deficient mice received DSS for 7 days (Figure 2a). Following the induction of colitis, a significant reduction in body weight loss (Figure 2b), disease activity index (Figure 2e), and colitis histology index score (Figure 2f) was observed in miR-511-deficient mice compared to WT mice. However, no differences were observed between the groups in terms of spleen weight (Figure 2c) and colon weight/length ratio (Figure 2d). Representative histology pictures of the colons of WT and miR-511-deficient mice under DSS conditions are shown in Figure 2g, which shows less inflammation in the miR-511-deficient mice comparatively. Also, a significant downregulation of colonic cytokine and chemokine levels (TNF-α, IL-6, MCP-1, and CXCL10) was observed in miR-511-deficient mice compared to WT mice (Figure 2h). These data indicate that miR-511 deficiency ameliorates DSS-induced colitis in mice. 

### 3.3. Changes in Colonic Monocyte and Maturing Monocyte Populations in miR-511-Deficient Mice in Inflamed Condition 

**Figure 3:**
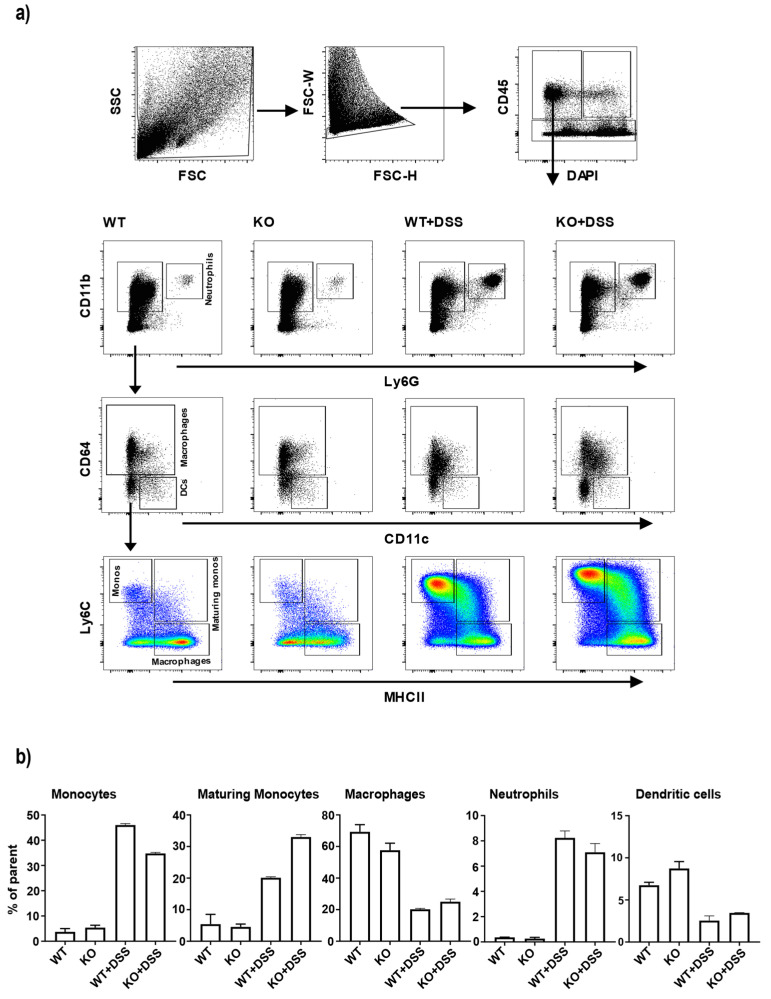
Immune cell composition in the colons of wild-type and miR-511-deficient mice. Immune cell populations of colons from WT and miR-511-deficient mice were analyzed using flow cytometry. The following gating strategy was used: (**a**) Debri was excluded using forward scatter (FSC) and side scatter (SSC) gating, followed by excluding doublets by FSC-H and FSC-W gating. Live leukocytes were selected as DAPI-CD45+. The resulting cells were then analyzed for CD11b, Ly6G, CD11c, CD64, Ly6C, and MHCII to identify neutrophil, DC, monocyte (monos), maturing monocyte (maturing monos) and macrophage populations. (**b**) Relative contribution of different immune cell populations in control (WT, KO) and DSS groups (WT+DSS, KO+DSS), respectively. Neutrophil (CD11b+Ly6G+), dendritic cell (CD11b+Ly6G-CD64-CD11c+), monocyte (CD11b+Ly6G-CD64+Ly6C+MHCIIa-), maturing monocyte (CD11b+Ly6G-CD64+Ly6C+MHCIIa+), macrophage (CD11b+Ly6G-CD64+Ly6C-MHCIIa+) contributions are indicated as percentage of parent gate. These data represents pooled data of two independent experiments with 5 mice per group per experiment. Error bars indicate standard error of the mean.

To further investigate the effect of miR-511 deficiency on immune cell composition in the colon tissue, we next examined subpopulations of lamina propria mononuclear phagocytes and neutrophils in colons with and without inflammation. Using flow cytometry we distinguished CD11b+Ly6G+ neutrophils, CD11b+Ly6G-CD64-CD11c+ dendritic cells (DCs), CD11b+Ly6G-CD64+Ly6C+MHCIIa- monocytes, CD11b+Ly6G-CD64+Ly6C+MHCIIa+ maturing monocytes and CD11b+Ly6G-CD64+Ly6C-MHCIIa+ macrophages, through distinctive antibody panels as established earlier [41,42] (Figure 3a). As previously described [41,42], healthy colons compared to inflamed colons contained relatively more DCs and mature macrophages while in inflammatory conditions an increase in monocytes, maturing monocytes and neutrophils were seen (Figure 3b). In healthy colons, no significant differences were found in relative contribution of the analyzed populations between WT and miR-511-deficient mice (Figure 3b). This suggests that miR-511 deficiency does not affect resident myeloid populations in healthy state. Interestingly, in inflammatory conditions colonic mononuclear phagocyte populations were differently distributed in miR-511-deficient mice compared to WT control mice (Figure 3b). Comparatively, miR-511-deficient mice showed lower percentage of monocytes and a higher percentage of maturing monocytes present in inflamed colons while no difference was seen for neutrophils, DCs and macrophage populations. The higher number of monocytes is most likely a reflection of differences in severity of colonic inflammation between miR-511-deficient and WT mice.

### 3.4. miR-511 Deficiency Affects Wdfy1

**Figure 4:**
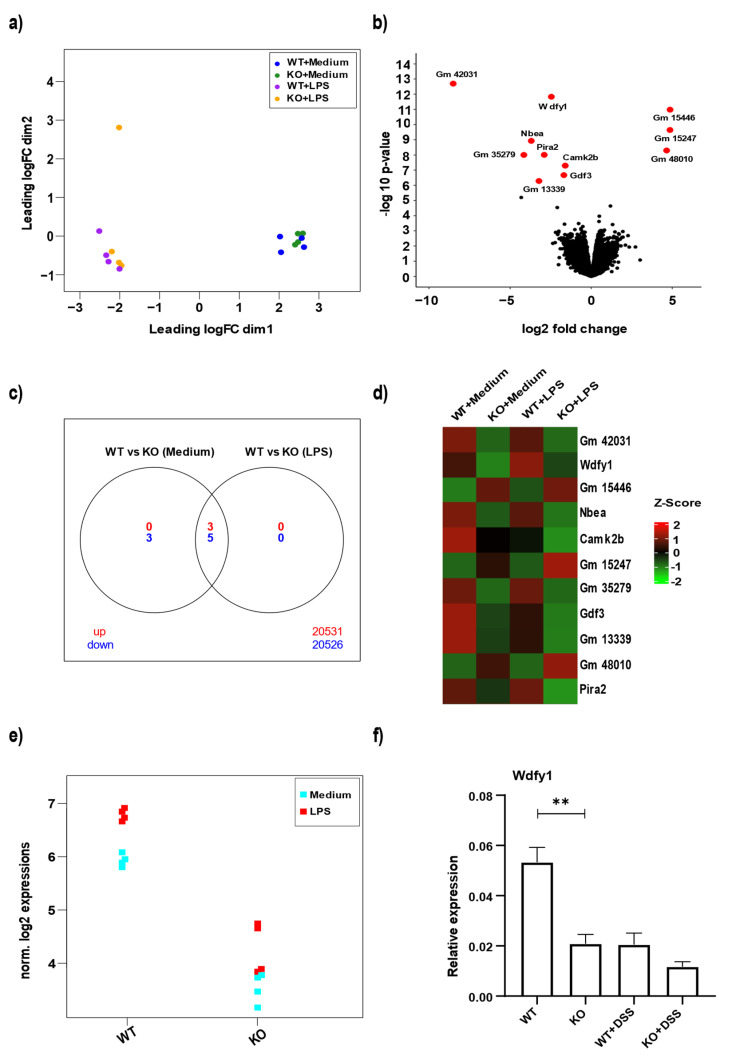
Differential gene expression analysis of miR-511-deficient and wild-type macrophages. Bone marrow-derived M2 macrophages were generated from WT and miR-511-deficient mice. WT and miR-511-deficient groups (KO) with and without LPS stimulation were compared. (**a**) Multidimensional scaling plot (MDS) using the top 500 most variable genes between samples to calculate the pairwise distances approximating the typical log2 fold changes between samples. The 16 samples are designated as WT+medium (blue), KO+medium (green), WT+LPS (purple), and KO+LPS (orange). (**b**) Volcano plot comparing WT with miR-511-deficient conditions. Differentially transcribed genes with a −log10 (*p*-value) > 6 are indicated with a red dot and annotated by their gene symbol. (**c**) Venn diagram of significant differentially expressed genes, upregulated genes (red) and downregulated genes (blue). A *p*-value <0.05 corrected for multiple testing using the Benjamini-Hochberg false discovery rate is used as a cut-off (**d**) Heatmap of top 11 differentially expressed genes. The mean of the gene expression per condition was used and the values were standardized. (**e**) Wdfy1 transcription levels in WT and miR-511-deficient macrophages with LPS (designated in red) and without LPS stimulation (designated in blue). (**f**) Wdfy1 gene expression in colon homogenates of control (WT, KO) and DSS groups (WT+DSS, KO+DSS), respectively by qPCR. N = WT = 4, KO = 5, WT+DSS = 9, KO+DSS = 10. The mRNA levels were normalized for their reference genes. Individual values are expressed as mean and standard error of mean. Statistical differences were tested by independent *t*-test, where a *p*-value of <0.05 was considered to be significant. ** *p*-value < 0.01.

In order to investigate potential miR-511 targets in macrophages, we screened for differentially expressed genes using RNA-seq. M2 macrophages were generated from WT and miR-511-deficient mice and groups with and without LPS stimulation were compared. To visualize the variation in expression between samples, a multidimensional scaling plot (MDS) was demonstrated, where it became apparent that miR-511 deficiency had little effect on macrophage gene expression (Figure 4a). As expected, LPS stimulation had a clear significant effect on macrophage gene expression in both WT and miR-511-deficient macrophages (Figure 4a). When comparing WT and miR-511-deficient macrophages only 11 genes were differentially expressed (Figure 4b,d) of which 3 genes were upregulated and 5 genes were downregulated in both groups (controls and LPS stimulated macrophages) whereas 3 genes were downregulated (growth differentiation factor 3, calcium/calmodulin-dependent protein kinase II beta and an unidentified gene 13339) in control groups alone as seen in the Venn diagram (Figure 4c). Furthermore, in the group of differential expressed genes, Wdfy1 was the only gene that was identified by TargetScan as a potential target of miR-511-3p in mice (fold change and BH-significance) (Figure 4e). WDFY1 is a protein involved in positive regulation of the TLR3 and TLR4 signaling pathways [20]. To investigate this, we next checked for colonic Wdfy1 gene expression and also observed a significant downregulation of Wdfy1 mRNA in colons of miR-511-deficient mice compared to WT (Figure 4f). Interestingly, DSS induced inflammatory conditions reduced Wdfy1 expression in both WT and miR-511-deficient mice (Figure 4f). Also, less Wdfy1 protein was detected in the miR-511 deficient mice compared to controls with no significant difference observed between the groups in DSS condition (supplementary Figure S2a). A representative immunohistochemistry picture is shown in supplementary Figure S2a. Moreover, WDFY1 protein was also detected in the colons of non-inflammatory bowel disease (IBD) controls and inflamed and non-inflamed colon section of IBD patients (supplementary Figure S2b). WDFY1 is localized to endosomal membrane as reported earlier [43], and interestingly, from our staining it seems that WDFY1 is present in both endosomal membrane and nucleus in mouse and human colonic crypts, although no clear distinction can be made in between the healthy and IBD colon sections. Together our data underscore that Wdfy1 is one of the targets of miR-511 involved in macrophage inflammatory responses. 

### 3.5. Macrophages from miR-511-Deficient Mice Have Reduced Wdfy1 Expression, Which Affects Antimicrobial Responses 

**Figure 5:**
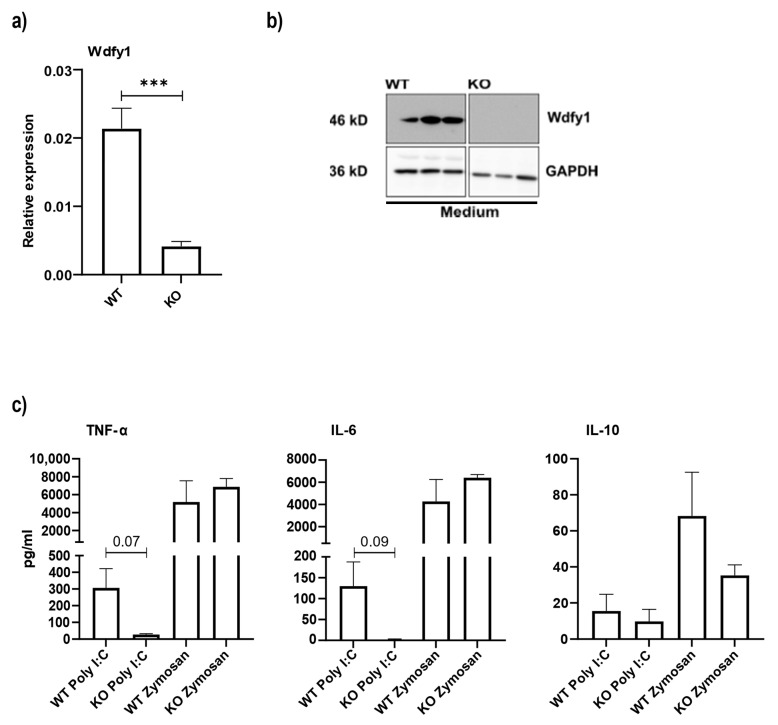
Role of Wdfy1 in affecting antimicrobial responses. (**a**) Wdfy1 gene expression in bone marrow-derived M2 macrophages by qPCR. mRNA levels were normalized for their reference genes. N = 5 WT and 5 miR-511-deficient mice. (**b**) Wdfy1 protein expression in M2 differentiated BMDMs in triplicates per group as seen by western blot. N = 3 WT and 3 miR-511 deficient (**c**) Cytokine response (TNF-α, IL-6 and IL-10) by BMDM after Poly I:C and Zymosan stimulation by CBA. N = 3 for both groups. Individual values are expressed as mean and standard error of the mean. Statistical differences were tested by independent *t*-test, where a *p*-value of <0.05 was considered to be significant. *** *p*-value < 0.001.

We further investigated Wdfy1 protein levels and anti-microbial responses in WT and miR-511-deficient macrophages. M2 bone marrow-derived macrophages were generated and as expected a significant reduction in Wdfy1 mRNA expression was observed in miR-511-deficient macrophages (Figure 5a). Surprisingly when determining Wdfy1 protein by western blotting, Wdfy1 protein was not detected in miR-511-deficient macrophages (Figure 5b). We had already found that LPS mediated cytokine responses were reduced in miR-511-deficient macrophages compared to WT macrophages (Figure 1d). To determine if responses via other pattern recognition receptors are also affected in these macrophages we next challenged macrophages with Polyinosinic:polycytidylic acid (Poly I:C) (TLR3 agonist) and Zymosan (dectin-1 and TLR2 agonists). Interestingly, Poly I:C induced TNF-α and IL-6 responses were reduced in miR-511-deficient macrophages compared to WT macrophages and miR-511 deficiency did not affect macrophage cytokine responses towards Zymosan (Figure 5c). Together these data suggest that miR-511 specifically regulates TLR3 and TLR4 responses via regulation of Wdfy1 but does not affect other pattern recognition receptors like TLR2 and dectin-1, which are involved in responses towards Zymosan [44,45].

## 4. Discussion

miR-511 is embedded within intron region 5 of the CD206 gene (MRC1) [5,6]. Previously, we showed that CD206 deficient mice also lacked miR-511 and that this knockdown of miR-511 led to downregulation of TLR4 and reduced intestinal inflammation in these mice [16]. In this study, we confirm, as hypothesized before, that miR-511 deficiency protects against DSS colitis (Figure 2). Reduced colonic inflammation in miR-511 deficient mice coincided with a lower monocyte percentage and a higher maturing monocytes percentage in inflamed colons compared to WT (Figure 3b). Monocytes are recruited to sites of infection and inflammation where they mediate antimicrobial activity, thus playing a key role in defense against pathogens [46,47]. In our study, fewer monocyte populations were recruited in miR-511-deficient colons, which corroborates with reduced intestinal inflammation (Figure 3b). 

Previously we showed that CD206-deficient mice lacking miR-511 affected macrophage TLR4 responses [16]. Other studies have also investigated the role of miR-511 in TLR4 signaling [5,17]. For instance, miR-511 knockdown leads to enhanced TLR4 protein levels in differentiating dendritic cells [5]. In this present study, miR-511-deficient mouse macrophages demonstrated reduced pro-inflammatory cytokine responses (TNF-α and IL-6) upon TLR4 stimulation with LPS (Figure 1d). This also corroborates with our previously reported result [16]. 

This study is the first to identify Wdfy1 as a potential target for miR-511. Using RNA-seq to identify potential targets on bone marrow-derived M2 macrophages, we found Wdfy1 to be one of the few differentially expressed genes between WT and miR-511-deficient macrophages (Figure 4). WDFY1 is a crucial adapter protein involved with positively regulating TLR 3 and 4 signaling pathways, by recruiting TIR-domain-containing adapter-inducing interferon-β (TRIF) [20]. Furthermore, WDFY1 was also shown to play a role in autophagy [48,49] and Wdfy1 expression is also related to mitochondrial dysfunction in Alzheimer’s disease of a mouse model [50]. For instance, an increase in WDFY1 levels followed by vascular endothelial growth factor-nontyrosine kinase receptor (VEGFC-NRP2) axis depletion induces cell death [48]. Although the function of WDFY1 still remains obscure, one study reported the role of Wdfy1 in innate immunity by comparing the immune response of WT and Wdfy1-deficient mice against TLR3 (Poly I:C) and TLR4 (LPS) agonists [21]. Wdfy1-deficient mice showed impaired inflammatory cytokines in BMDMs [21], which shows that mouse Wdfy1 is important for TLR3 and TLR4 signaling. In line with this, we first showed that the Wdfy1 gene was significantly downregulated in both colons of miR-511-deficient mice (Figure 4f) and macrophages cultured from these mice (Figure 5a). Interestingly, Wdfy1 was not detected at protein level in these macrophages (Figure 5b). Also, the mRNA expression of the gene in macrophages was low (Figure 5a) so it might indicate that miR-511 is protecting it against degradation and thus affecting stability of mRNA, post-translationally [51]. Besides reduced inflammatory responses upon TLR4 stimulation, miR-511-deficient macrophages also showed reduced TNF-α and IL-6 responses when stimulated with TLR3 agonist (Poly I:C) (Figure 5c). Upon Zymosan challenge, miR-511-deficient macrophages did not respond differently from WT macrophages, which suggests that signaling pathways downstream of Wdfy1 is affected while other pattern recognition remain intact (Figure 5c). Together this suggests that miR-511 deficiency causes a downregulation of Wdfy1 which affects TLR3 and TLR4 responses. However, to confirm a direct interaction between miR-511 and Wdfy1, experiments such as reporter gene assays or pull-down assays are needed. 

We are the first to report a protective effect of miR-511 deficiency against DSS-induced inflammation in mice. Our data suggests miR-511 regulates Wdfy1 expression which subsequently affects TLR (3 and 4) responses. The distinct role of miR-511 in inflammation needs to be unraveled to understand disease pathogenesis and mechanisms in IBD better. Further studies are required to explore the function and interaction of miR-511 and WDFY1 in humans. 

## Figures and Tables

**Table 1:** Primer sequences used in qPCR reactions.

Gene	5′-Forward Sequence	5′-Reverse Sequence
GAPDH	ATGTGTCCGTCGTGGATCTGA	ATGCCTGCTTCACCACCTTCT
Cyclophilin	ATGGTCAACCCCACCGTGT	TTCTGCTGTCTTTGGAACTTTGTC
EEF2	TGTCAGTCATCGCCCATGTG	CATCCTTGCGAGTGTCAGTGA
RPLP0	CCAGCGAGGCCACACTGCTG	ACACTGGCCACGTTGCGGAC
TNF-α	AAAGCATGATCCGCGACGT	TGCAAGCAGGAATGAGAA
IL-6	GAGTTGTGCAATGGCAATTCTG	TGGTAGCATCCATCATTTCTTTGT
IL-10	TGTCAAATTCATTCATGGCCT	ATCGATTTCTCCCCTGTGAA
CXCL10	CCAAGTGCTGCCGTCATTTTC	TCCCTATGGCCCTCATTCTCA
MCP-1	AGGCTGGAGAGCTACAAGAGGAT	TCTCATTTGGTTCCGATCCAGG
TLR4	TGTCATCAGGGACTTTGCTG	TGTTCTTCTCCTGCCTGACA
CD206	TGTGGTGAGCTGAAAGGTGA	CAGGTGTGGGCTCAGGTAGT
Wdfy1	AGAGTGCAGTCACTGTGCTACC	CTGCTCACACTTCTGACAGGAG

## Data Availability

Data available on request.

## Supplementary Materials

The following are available online at https://www.mdpi.com/article/10.3390/cells11010058/s1, Figure S1: Relative mRNA expression of cytokines in BMDMs stimulated with LPS. (a) mRNA expression levels of TNF-α, IL-6 and IL-10 after 24 h of LPS stimulation. The levels of mRNA were normalized for reference genes. N = 5 WT and 6 miR-511-deficient mice. Individual values are expressed as mean and standard error of mean. Statistical differences were tested by independent t-test, where a *p*-value of <0.05 was considered to be significant. Figure S2: Detection of WDFY1 protein in colon sections of mouse and human IBD sections. (a) Detection of Wdfy1 by immunohistochemistry in colon tissue in WT and miR-511-deficient mice in control and DSS conditions, 10× magnification. (b) Detection of WDFY1 by immunohistochemistry in colon tissue sections in non-IBD healthy and IBD inflamed and non-inflamed colon sections, 10× magnification.
