# Supplementary figures and images for: miR-511 Deficiency Protects Mice from Experimental Colitis by Reducing TLR3 and TLR4 Responses via WD Repeat and FYVE-Domain-Containing Protein 1
Source: Cells. 2021 Dec 25;11(1):58. doi: 10.3390/cells11010058 (PMC8750561; doi:10.3390/cells11010058)

a)

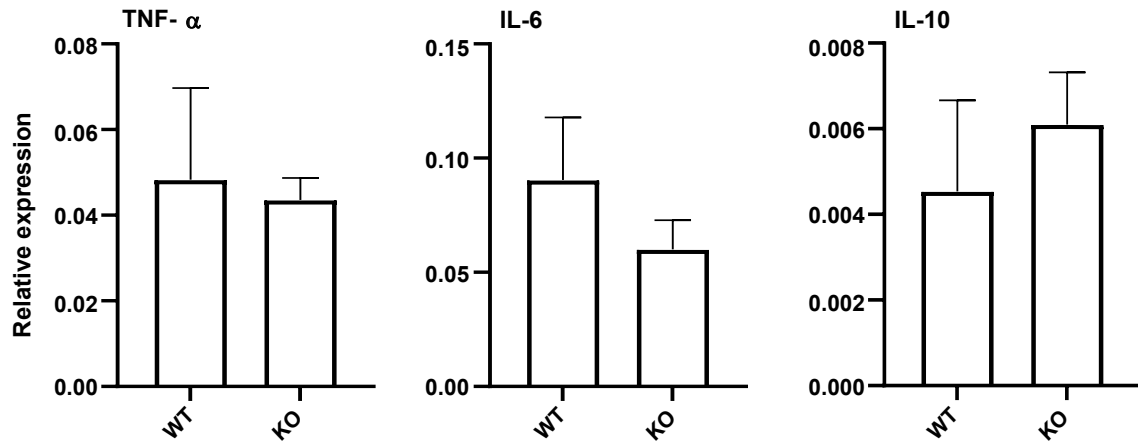

a)

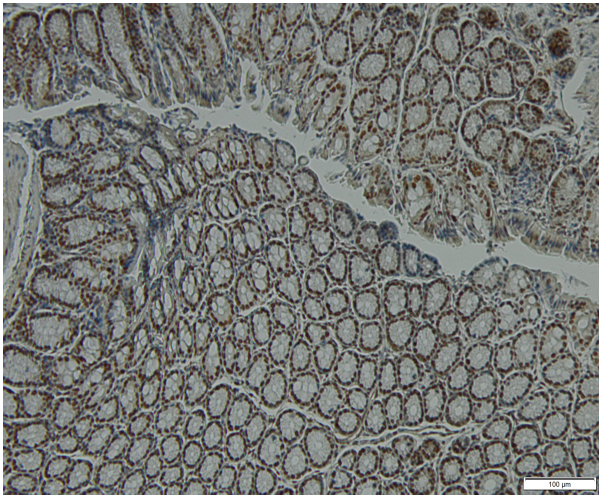

WT

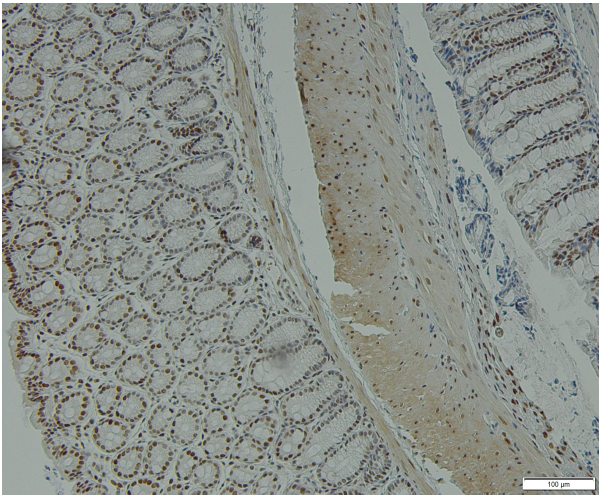

KO

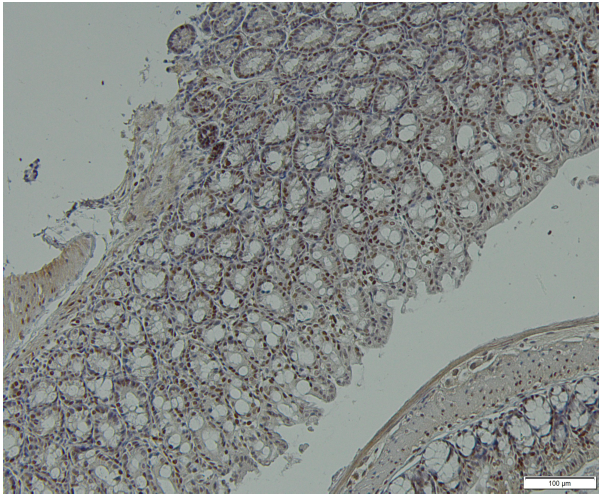

WT+DSS

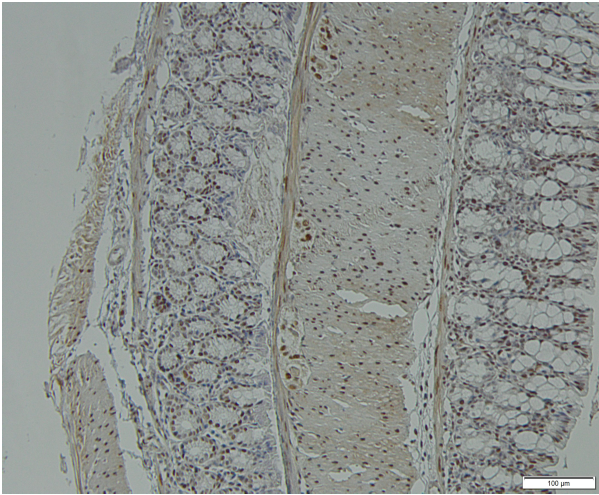

KO+DSS

b)

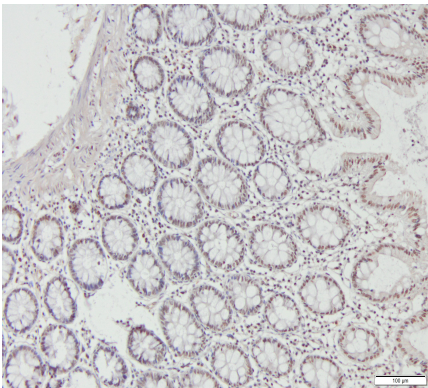

Healthy control

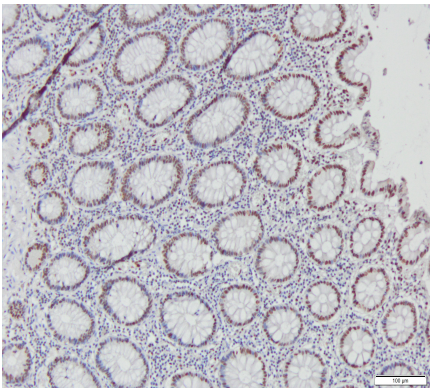

Non-inflamed

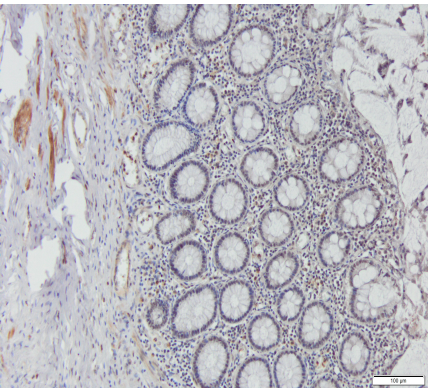

Inflamed

Supplement: Supplementary file 1 [file cells-11-00058-s001.zip › cells-1491531-supplementary.pdf]
